# Assessment of immediate production impact following attenuated PRRS type 2 virus vaccination in swine breeding herds

**DOI:** 10.1186/s40813-019-0120-2

**Published:** 2019-06-11

**Authors:** Cesar A. A. Moura, Clayton Johnson, Samuel R. Baker, Derald J. Holtkamp, Chong Wang, Daniel C. L. Linhares

**Affiliations:** 1 Veterinary Diagnostic and Production Animal Medicine Department, Iowa State University, 1809 S Riverside Dr, Ames, Iowa 50011 USA; 2 Carthage Veterinary Services, Inc, 303 North 2nd Street, 62321 Carthage, IL USA

**Keywords:** Swine, PRRS, Vaccination, MLV, Outbreak, Epidemiology

## Abstract

**Background:**

To mitigate production impact of porcine reproductive and respiratory syndrome (PRRS) virus outbreaks, it has been common to preventively vaccinate swine breeding herds using PRRS modified live virus (MLV) vaccine. However, attenuated PRRS virus (PRRSv) may result negative impact on farm productivity. The objective of this study was to measure the immediate impact of PRRS type 2 MLV vaccine on breeding herd performance under field conditions. Eight PRRS-stable farms routinely mass vaccinating females with commercial PRRS MLV vaccines were enrolled on study. Vaccination dates were collected and weekly changes in abortions, neonatal losses, pre-weaning mortality, pigs weaned per sow, and wean-to-first-service interval were assessed for up to 6 weeks after each vaccination. A 6-week period prior to each vaccination was established as baseline. Statistical process control (SPC) analysis was conducted to detect significant productivity decreases after MLV interventions, on each farm, and a mixed regression model was used, at the aggregated data level, to assess the productivity change 6 weeks after PRRS MLV vaccinations, compared to baseline.

**Results:**

Out of 65 herd-MLV vaccinations, SPC analysis detected increase on abortions 4 times (6.1%), on neonatal losses 7 times (10.7%), on pre-weaning mortality 2 times (3%), on wean-to-first-service interval 2 times (3%), and no change in total pigs weaned. On aggregated data analysis, there was no significant change in abortion rate, neonatal losses, number of pigs weaned per sow, and wean-to-first-service interval. However, there was an increase of 0.26% of pre-weaning mortality 2 weeks after vaccination compared to the baseline.

**Conclusions:**

Under study conditions, individual PRRS-stable sow farms had experienced transient, and numerically small changes in productivity following PRRS type 2 MLV vaccination. There was a small increase of pre-weaning mortality 2 weeks after vaccination, but no evidence of significant production impact at aggregated data analysis for abortion rate, neonatal losses, pigs weaned per sow and wean-to-first-service interval.

## Background

Porcine reproductive and respiratory syndrome virus (PRRSv) continues to significantly increase the cost of pig production due to reproduction losses and reduced growth performance [1–3]. To reduce losses due to PRRSv infection, veterinarians have implemented practices to prevent virus introduction (i.e. biosecurity), as well as developed strategies to control, and/or eliminate the virus from individual pig herds and from regions [4–6]. Most PRRSv control/elimination efforts consist of decreasing (or eliminating) virus replication in the herd based on a combination of strategic PRRSv immunization and changes in pig flow targeting reduction in within-herd virus transmission [7]. Immunization of US swine populations against PRRSv has been accomplished in large part with the use of modified-live (attenuated) virus vaccine (MLV) or field virus inoculation (FVI) [8].

The American Association of Swine Veterinarians (AASV) proposed a standardized terminology for communicating the PRRS status of breeding herds [9]. Briefly, upon infection herds are classified as “positive unstable”. When there is the failure of PRRSv RNA detection by RT-PCR in due-to-wean piglets for at least 90 consecutive days, the herd is classified as ‘positive stable’. Then, when there is no evidence of PRRSv circulation in the breeding herd, as demonstrated by lack of PRRS-associated clinical signs, and incoming gilts remaining serologically negative for PRRSv, the herd is defined as “provisional negative”. Finally, when there is no virus circulation, and no anti-PRRSv antibody circulation in the population, the herd is defined as PRRS-negative (or naïve).

Field studies have compared exposure programs to control PRRSv in sow herds, in terms of time to produce PRRSv-negative piglets at weaning, and/or production losses following the outbreak [10, 11]. One important reported finding was that herds with recent history of PRRSv exposure achieved stability (i.e. failure to detect PRRSv RNA in due to wean piglets consistently for 3 consecutive months testing 30 piglets by RT-PCR), and recovered productivity significantly sooner than herds without recent history of PRRSv infection. Another study approached the question of the economic benefit of practicing preventive vaccination using attenuated virus vaccine as an attempt to “build” anti-PRRSv immunity prior to outbreak with wild type strains [12]. It was demonstrated that in the one hand vaccination increases herd immunity and reduces time-to-stability and impact on productivity when the herd becomes infected with wild type viruses. On the other hand, preventively vaccinating a breeding herd also increases production costs (vaccine costs) and potentially attenuated PRRSv from vaccines has a negative impact on farm productivity [13–15]. The study documented that it was economically beneficial to preventively vaccinate breeding herds whenever the expected outbreak frequency was less than every 2.1 years. However, the “2.1 year” mark is highly sensitive to the attributed “negative impact” of MLV on PRRS-negative or PRRS-stable breeding herds. The authors of that study, due to the scarce availability to studies documenting safety of contemporary attenuated PRRSv vaccines, used a conservative approach assuming that preventive use of PRRS MLV vaccines resulted in decrease of 1 piglet per sow per year in the breeding herd productivity. Thus, there is the need to better define the production impact on PRRS-stable breeding herds adopting PRRS MLV vaccination.

The objective of this epidemiological study was to measure the immediate impact of PRRS type 2 MLV on key breeding herd performance parameters using a natural experiment under field conditions. This information will provide information to best feed the existing economic models to assist swine veterinarians to take informed decisions regarding the use of PRRS MLV vaccine as a preventive tool.

## Methods

### Study design

**Fig. 1:**
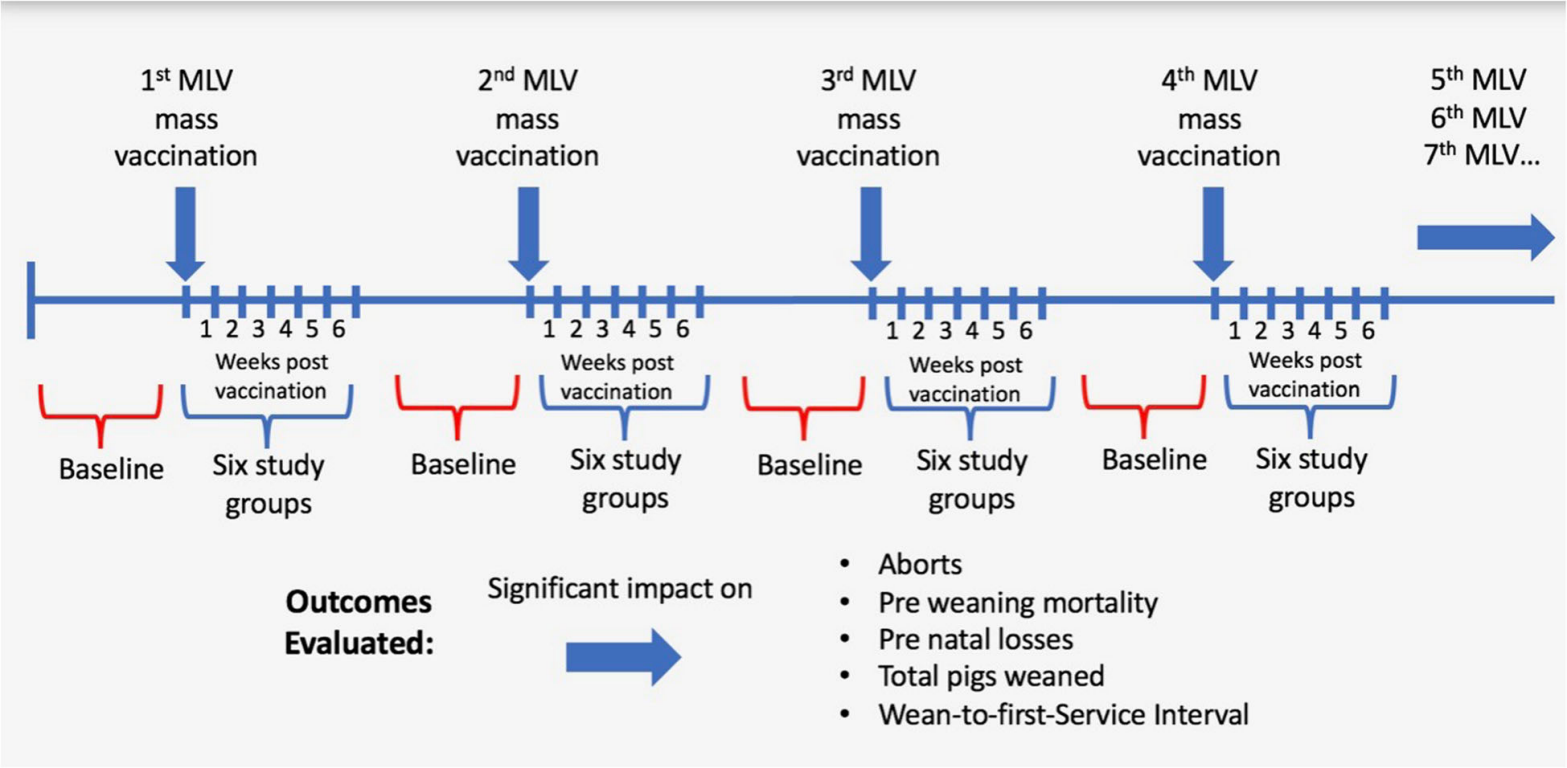
Study design

This was a retrospective epidemiological study to evaluate the immediate impact of PRRS MLV vaccination on the breeding herd productivity. Eight sow farms from a production system in the USA, classified as PRRS-stable according to the American Association of Swine Veterinarians classification [9], that adopted routinely vaccinations using commercially available PRRS type 2 MLV vaccines were conveniently identified, and enrolled in the study. Study herds adopted a whole-herd vaccination program, where all breeding stock (sows and gilts) in the farm were exposed to one intramuscular dose (recommended by the fabricant) of MLV regardless of age, parity, or stage of production (lactating, gestating, weaned). Statistical models were used to assess changes in production parameters on six weeks following PRRS MLV vaccination events, compared to 6 weeks before the interventions (Fig. 1). It was conducted two types of analysis: a) a herd-level analysis, where statistical process control (SPC) was implemented on each parameter per herd, and b) an aggregated analysis, which combined information from all herds. The period of 6 weeks following vaccination was chosen to represent the expected duration of the viremic phase following infection of individual sows pre-exposed to PRRSV. This study was based on retrospective data, and hence no animal care and use approval was required.

### Production parameters, and vaccination dates

The following production parameters were recorded from the study herds, in a Microsoft Excel spreadsheet: number of abortions, defined as the counts of sows that aborted at least one fetus; pre-weaning mortality, defined as the number of piglets that were born alive, and died before weaning; neonatal losses, defined as the difference of average number of pigs born per litter and number of pigs born alive per litter; total pigs weaned, defined as the total number of pigs weaned; and wean-to-first-service interval, defined as the average number of days between weaning and first service. All parameters were recorded on a weekly interval during the study period. The vaccination dates for each study herd were also collected on a Microsoft Excel spreadsheet.

### Herd enrollment, eligibility and exclusion criteria

Breeding sow farms were eligible for this study if they were a) diagnostic evidence to support the status of ‘PRRS stable’ according to the AASV guidelines, b) implementing routine vaccinations with a commercial PRRS MLV vaccine (PRRS Ingelvac MLV (Boehringer Ingelheim Vetmedica Inc., St Joseph, MO); PRRS Ingelvac ATP (Boehringer Ingelheim Vetmedica Inc., St Joseph, MO); or Fostera PRRS (Zoetis Inc., Parsippany-Troy Hills, NJ)), c) able to provide vaccination dates, and weekly production records required for this study, and d) not having clinical outbreaks of infectious diseases that may impact the parameters that were measured, including porcine epidemic diarrhea (PED).

### Statistical analysis

A PROC MACONTROL procedure of SAS 9.4 (SAS Institute, Inc., Cary, NC) was used to perform a descriptive analysis, based on statistical process control (SPC), of the data on the individual farm level to detect negative impact on productivity up to 6 weeks after each MLV intervention, compared to a baseline period of 21 weeks before the first vaccination of the herd. The exponential weighted moving average (EWMA) was chosen as a SPC method and set with 3 sigma (which adjusts the width of the upper and lower control limits [16]), and a 0.4 lambda constant (which represents the weight assigned to the most recent sample mean included [16]). A decrease in productivity, after up to six weeks following each MLV intervention, with performance parameters crossing the EWMA control limits, was defined as a signal. More specifically, an increase of abortion rate (percentage of sows in the herd inventory reported with abortion event), pre-weaning mortality (percentage of dead piglets from total pigs born), neonatal losses (difference of total number of piglets born and piglets born alive), or wean to first service interval; or a decrease in total pigs weaned. The frequency of significant changes in productivity following MLV vaccination, as well as the magnitude and duration of these changes were reported.

Moreover, it was conducted analysis with the aggregated data from all herd-vaccinations to assess the ‘production system effect’ of MLV vaccination on sow farm productivity. The PROC GLIMMIX procedure of SAS 9.4 (SAS Institute, Inc., Cary, NC) was used to build a mixed regression model to assess change of each production parameter up to 6 weeks following the reported PRRS MLV vaccination date, compared to a 6-week period immediately before vaccination. Poisson distribution was used for abortions, pre-weaning mortality and number of pigs weaned per week, since those responses were defined as counts. A log-link function was used in the Poisson models, and an offset variable was used to adjust the analysis for herd size, controlling abortions by the average sow inventory of the week, pre-weaning mortality by the total number of pigs born on the week, and total pigs weaned by the number of sows weaned on the week. For neonatal losses, we used a log-normal distribution, because it was the difference between two means. Exponential distribution with a log-link function, was used for the wean-to-first-service interval, since it was a time to event response. Moreover, herd was used as random effect, and weeks after MLV vaccination was used as a categorical fixed effect for all the models. A level of significance of *P* < 0.05 was used to compare the productivity of each week after the MLV intervention (*n* = 6) to the baseline, with a one-sided *p*-value detecting only if the change represented a negative impact on performance. SAS 9.4 (SAS Institute, Inc., Cary, NC) was used to conduct the Bonferroni adjustment for multiple comparisons on the parameters that we found to be significant, by including the ‘adjust = BON’ in the LSMEANS statement from the PROC GLIMMIX model.

## Results

There were 65 eligible herd-vaccinations, and 4 non-eligible herd-vaccinations. The reason for non-eligibility were: PRRSV outbreaks causing herd instability (*n* = 4). The median number of vaccinations per herd was 8, with minimum of 5 and maximum of 11. The median period between vaccinations was 13 weeks, with the minimum value of 4 and maximum of 41. There were 780 production record weeks evaluated.

On the SPC analysis, which described the herd-level production losses following each of the 65 MLV vaccinations, there were signals in abortions following 4 herd-vaccinations, a significant increase in neonatal losses after 7 herd-vaccinations, significant increase in pre-weaning mortality after 2 herd-vaccinations, and increase in wean-to-first-service interval after 2 herd-vaccinations. There was no significant change on total pigs weaned after MLV interventions (Table 1).

**Table 1:** Statistical process control (SPC) analysis of the data on the individual farm level

Production parameter	Frequency of signals^a^	Duration of signals	Magnitude of signals
Abortion rate	4/65 (6.1%)	1 to 7 weeks	0.11 to 0.16%
Neonatal losses	5/65 (7.7%)	1 to 5 weeks	0.34 to 1.85
Pre-weaning mortality	2/65 (3.1%)	1 to 5 weeks	3.99 to 5.21%
Total pigs weaned	0/65 (0.0%)	N/A	N/A
Wean-to-first-service interval	2/65 (3.1%)	3 to 5 weeks	1.03 to 1.44 days

^a^ = A decrease in productivity following each MLV intervention, with performance parameters crossing the EWMA control limits, was defined as a signal

There was no significant negative effect of PRRS MLV vaccination on the aggregated data of all herd vaccinations for abortion rate (Table 2), neonatal losses (Table 3), pigs weaned per sow (Table 4), or wean to first service interval (Table 5) in any of the 6 weeks post vaccination compared to the baseline period. However, there was an increase of 0.26% of pre-weaning mortality on the week 2 after vaccination compared to the baseline (Table 6).

**Table 2:** Means of abortion rate over time, following 65 herd vaccinations, compared to baseline

Weeks post PRRSv MLV vaccination	Abortion rate means	Standard Error of the mean	*P*-value*
Baseline	0.069%	0.000116	–
1	0.073%	0.000129	0.1658
2	0.070%	0.000124	0.4082
3	0.076%	0.000133	0.0775
4	0.068%	0.000119	0.6336
5	0.070%	0.000124	0.4148
6	0.068%	0.000120	0.6193

* = mixed regression model of each group compared to baseline

**Table 3:** Means of neonatal losses over time, following 65 herd vaccinations, compared to baseline

Weeks post PRRSv MLV vaccination	Neonatal losses means	Standard Error of the mean	*P*-value*
Baseline	0.979	0.05385	–
1	0.979	0.05933	0.4978
2	0.978	0.05933	0.5211
3	0.990	0.05933	0.3569
4	0.997	0.05933	0.2779
5	0.958	0.05952	0.7609
6	0.999	0.05952	0.2567

* = mixed regression model of each group compared to baseline

**Table 4:** Means of pigs weaned per sow over time, following 65 herd vaccinations, compared to baseline

Weeks post PRRSv MLV vaccination	Pigs weaned per sow means	Standard Error of the mean	*P*-value*
Baseline	10.49	0.1338	–
1	10.74	0.1389	1.000
2	10.50	0.1359	0.5920
3	10.49	0.1358	0.5480
4	10.52	0.1361	0.9046
5	10.81	0.1399	1.000
6	10.64	0.1377	1.000

* = mixed regression model of each group compared to baseline

**Table 5:** Means of wean to first service interval over time, following 65 herd vaccinations, compared to baseline

Weeks post PRRSv MLV vaccination	Wean-to-first-service interval means	Standard Error of the mean	*P*-value*
Baseline	5.82	0.3121	–
1	5.87	0.7289	0.4727
2	5.88	0.7305	0.4665
3	5.86	0.7272	0.4796
4	5.81	0.7217	0.5021
5	5.83	0.7357	0.4915
6	5.72	0.7213	0.5490

* = mixed regression model of each group compared to baseline

**Table 6:** Means of pre-weaning mortality over time, following 65 herd vaccinations, compared to baseline

Weeks post PRRSv MLV vaccination	Pre-weaning mortality means	Standard Error of the mean	*P*-value*
Baseline	13.97%	0.01497	–
1	13.12%	0.01407	1.000
2	14.23%	0.01526	0.0076
3	13.89%	0.01490	1.000
4	13.28%	0.01424	1.000
5	12.59%	0.01351	1.000
6	13.09%	0.01404	1.000

* = mixed regression model of each group compared to baseline

## Discussion

This study investigated the immediate effect of PRRS MLV vaccination on selected key production performance indicators. The ‘immediate’ effect was defined as a significant change on production parameters within 6 weeks after vaccination, as compared to 6 weeks prior to vaccination. As described in the materials and methods, the period of 6 weeks was chosen to represent the expected duration of the viremic phase following infection of individual sows that were pre-exposed to PRRSV. It was assumed that if PRRS MLV vaccination had a significant impact on the productivity parameters under field conditions, the change would have happened within the viremic phase of infection.

Under the conditions of this study, vaccinating for PRRS using MLV had no significant impact on abortion rate, neonatal losses, pigs weaned per sow, and wean to first service interval. The only significant production impact was an increase in pre-weaning morality of 0.26% on the week 2 post vaccination. This association of mass vaccination and increased mortality after two weeks is possibly related to the vaccine virus shedding and replication in the pigs. The reason for having significant differences on mortality and no impact on pigs weaned, is because the effect size of the difference was low. The impact of PRRS MLV vaccination on overall herd productivity was not significant on a system level, but there were some small transient changes in productivity on individual farms. As previously mentioned, this data provides information to best feed the existing economic models to assist swine veterinarians and swine producers to take informed decisions regarding the use of PRRS MLV vaccine as a preventive strategy.

The implementation of preventive vaccination of the breeding stock for PRRSv, with intent to minimize losses following wild-type virus introduction has been described [17, 18]. To the best of our knowledge, this is the first epidemiological study with multiple herd-vaccinations to document the effects of PRRS MLV vaccination on productivity parameters under field conditions.

Previous research documented that the impact of mass vaccination on overall breeding herd performance was 1 piglet per sow per year [12]. Results from this study suggests that the impact of mass vaccination on PRRS-stable breeding herds with type 2 PRRS MLV products is minimum and much lower than 1 piglet per sow per year. These results are supported by another research that reported no significantly clinical signs on pigs vaccinated, compared to the control non-vaccinated group [19].

Some negative effects of vaccinating pregnant sows for PRRS with a MLV vaccine have been reported and contrast the results of this study [13]. One study reported that the vaccination should be avoided on pregnant sows and implemented only on non-pregnant females, due to decreasing of the number of pigs born alive and weaned [14]. On another study, the same author reported that vaccinating sows against PRRSV caused production losses by increasing the number of stillbirths and mummified pigs [15].

Intrinsic factors from specific herds were in part adjusted for, by having the variable ‘Farm’ as random effect in the regression models. Season has been reported as a risk factor for PRRSv infection and related with changes in production parameters of breeding sows, such as abortions, neonatal losses, and pre-weaning mortality [20–23]. Thus, to take seasonality into account, this study had a dynamic baseline parameter for herd vaccination. More specifically, the effect of MLV vaccination on each production parameter was compared to the average of 6 weeks prior to vaccination, making the assessment of the effect of vaccinations robust to seasonal effects.

It is important to highlight that this study included only PRRS-stable sow farms (according to the American association of swine veterinarians criteria) [9], and thus results may not be applicable to PRRS-naïve, and/or positive-unstable PRRS sow farms. Moreover, the analysis was done for PRRSV type 2, and more studies should be conducted to extrapolate the results to PRRSV type 1. It was a limitation of the study the fact that it was retrospective, which makes it naturally subjected to recall and information bias.

## Conclusions

Although some farms had transient changes in productivity, at the aggregated data analysis level there was no significant change in abortion rate, neonatal losses, number of pigs weaned per sow, and wean to first service interval on PRRS-stable herds implementing PRRS MLV routinely vaccinations. For pre-weaning mortality, there was an increase of 0.26% on the week 2 after vaccination, compared to the baseline. According to the results of this study, adopting quarterly mass vaccinations may be a useful strategy to regularly immunize swine breeding herds to PRRSv.

## Abbreviations

AASV: American association of swine veterinarians
EWMA: Exponential weighted moving average
FVI: Field virus inoculation
MLV: Modified live virus
PRRS: Porcine reproductive and respiratory syndrome
PRRSV: Porcine reproductive and respiratory syndrome virus
RNA: Ribonucleic acid
RT-PCR: Reverse transcription polymerase chain reaction
SPC: Statistical process control
